# RNA silencing proteins and small RNAs in oomycete plant pathogens and biocontrol agents

**DOI:** 10.3389/fmicb.2023.1076522

**Published:** 2023-03-24

**Authors:** Edoardo Piombo, Bekele Gelena Kelbessa, Poorva Sundararajan, Stephen C. Whisson, Ramesh Raju Vetukuri, Mukesh Dubey

**Affiliations:** ^1^Department of Forest Mycology and Plant Pathology, Swedish University of Agricultural Sciences, Uppsala, Sweden; ^2^Department of Plant Breeding, Horticum, Swedish University of Agricultural Sciences, Lomma, Sweden; ^3^Department of Cell and Molecular Sciences, The James Hutton Institute, Dundee, United Kingdom

**Keywords:** *Phytophthora*, *Pythium*, *Lagenidium giganteum*, miRNA, sRNA, transposons, RNA silencing, biocontrol

## Abstract

**Introduction:**

Oomycetes cause several damaging diseases of plants and animals, and some species also act as biocontrol agents on insects, fungi, and other oomycetes. RNA silencing is increasingly being shown to play a role in the pathogenicity of *Phytophthora* species, either through trans-boundary movement of small RNAs (sRNAs) or through expression regulation of infection promoting effectors.

**Methods:**

To gain a wider understanding of RNA silencing in oomycete species with more diverse hosts, we mined genome assemblies for Dicer-like (DCL), Argonaute (AGO), and RNA dependent RNA polymerase (RDRP) proteins from *Phytophthora plurivora*, *Ph. cactorum*, *Ph. colocasiae*, *Pythium oligandrum*, *Py. periplocum*, and *Lagenidium giganteum*. Moreover, we sequenced small RNAs from the mycelium stage in each of these species.

**Results and discussion:**

Each of the species possessed a single DCL protein, but they differed in the number and sequence of AGOs and RDRPs. SRNAs of 21nt, 25nt, and 26nt were prevalent in all oomycetes analyzed, but the relative abundance and 5’ base preference of these classes differed markedly between genera. Most sRNAs mapped to transposons and other repeats, signifying that the major role for RNA silencing in oomycetes is to limit the expansion of these elements. We also found that sRNAs may act to regulate the expression of duplicated genes. Other sRNAs mapped to several gene families, and this number was higher in *Pythium* spp., suggesting a role of RNA silencing in regulating gene expression. Genes for most effector classes were the source of sRNAs of variable size, but some gene families showed a preference for specific classes of sRNAs, such as 25/26 nt sRNAs targeting RxLR effector genes in *Phytophthora* species. Novel miRNA-like RNAs (milRNAs) were discovered in all species, and two were predicted to target transcripts for RxLR effectors in *Ph. plurivora* and *Ph. cactorum*, indicating a putative role in regulating infection. Moreover, milRNAs from the biocontrol *Pythium* species had matches in the predicted transcriptome of *Phytophthora infestans* and *Botrytis cinerea*, and *L. giganteum* milRNAs matched candidate genes in the mosquito *Aedes aegypti*. This suggests that trans-boundary RNA silencing may have a role in the biocontrol action of these oomycetes.

## Introduction

RNA silencing is a form of gene regulation mediated by non-coding small RNAs (sRNAs), usually 18–32 nt in length (Hannon, 2002; Huang et al., 2019). RNA silencing is a conserved biological process involving three protein classes: Dicers or Dicer-like enzymes (DCLs), Argonaute proteins (AGOs) and RNA-dependent RNA polymerases (RDRPs). DCLs cleave long double stranded RNA (dsRNA) precursors (Bartel, 2009), producing double stranded sRNAs which are then unwound and loaded onto a protein complex called the RNA-induced silencing complex (RISC), where AGOs mediate the recognition of sequences complementary to sRNAs (Ma et al., 2018). Targeted mRNAs are cleaved by AGOs and degraded, or their translation is inhibited. RDRPs can amplify the silencing signal by driving production of additional sRNAs from the fragments of cleaved transcripts (Zong et al., 2009; Maniar and Fire, 2011). In some organisms, specific classes of AGOs can mediate the silencing of sRNA-complementary DNA sequences through methylation and other chromatin modifications (Holoch and Moazed, 2015).

Oomycetes are a group of organisms phenotypically similar to filamentous fungi but phylogenetically close to diatoms and brown algae (Gunderson et al., 1987; Thines, 2014). Many oomycetes are plant pathogens (Kamoun et al., 2015) and the most impactful of them is *Phytophthora infestans*, causal agent of potato late blight. This disease was the cause of the Irish famine in the nineteenth century and it continues to threaten potato production today (Haverkort et al., 2008). The genus *Phytophthora* also includes other pathogenic species, such as *Phytophthora cactorum* causing leather rot and crown rot on strawberries (Eikemo and Stensvand, 2015), *Phytophthora colocasiae* causing leaf blight of taro (Shakywar et al., 2013) and *Phytophthora plurivora* affecting roots in beech, oak and rhododendron (Jung et al., 2000; Jung, 2009; Lilja et al., 2011). Some oomycetes are also pathogens of animals, causing diseases on fish, crustaceans, and mammals (Phillips et al., 2008; Derevnina et al., 2016). Apart from the pathogens, certain oomycete species can be used as biocontrol agents (BCAs) against insects and plant pathogens. For example, *Pythium oligandrum* and *Pythium periplocum* can protect plants from fungal and oomycete pathogens and promote plant growth and fitness (Paul, 1999; Bělonožníková et al., 2022), while *Lagenidium giganteum* is a known parasite of the larvae of several mosquito species (Kerwin, 2007).

The genes involved in the RNA silencing mechanism have been identified in *Phytophthora* and their role in gene expression regulation was demonstrated (Vetukuri et al., 2011, 2012; Fahlgren et al., 2013; Qutob et al., 2013). Each of the *Phytophthora* spp. analysed to date possesses one DCL protein, at least four AGO proteins, and a single RDRP. *Ph. infestans* possesses five AGO proteins, divided into two clades (Vetukuri et al., 2011). Fahlgren et al. (2013) also identified a putative second DCL in *Phytophthora*, but this protein is more similar in sequence to Drosha proteins. Sequencing of sRNAs from *Phytophthora* spp. has revealed at least two major size classes (21 nt and 25/26 nt) of sRNAs (Vetukuri et al., 2012; Fahlgren et al., 2013; Qutob et al. 2013), although longer sRNAs of over 30 nt are also present (Vetukuri et al., 2012). Generation of sRNA size classes depends on their genomic locus of origin, as a higher proportion of 21 nt sRNAs mapped to inverted repeats and genes, presumably controlled at the post-transcriptional level, while sRNAs of 25–26 nt mapped predominantly to transposable elements and are suggested to control their transcription by inducing epigenetic modifications (Fahlgren et al., 2013; Jia et al., 2017). In addition, it was observed that silencing of specific elements of the RNA silencing pathway affects the production of sRNAs of precise sizes. For example silencing of *Dcl1* affected 21 nt sRNA production, while silencing of *Ago4* or *Ago5* affected 32 nt sRNAs, which seem to be generated in a DCL-independent manner (Vetukuri et al., 2012). Similarly, AGO1 is mostly associated with 21 nt sRNAs with a 5′ cytosine, while AGO4 binds mostly 25–26 nt sRNAs with a 5′ uracil (Åsman et al., 2016). Furthermore, the *Ph. infestans* clade 2 AGOs (AGO3, AGO4 and AGO5) do not have the typical catalytic amino acids in their PIWI domain, which normally mediates the cleavage of RNA (Nakanishi et al., 2012; Swarts et al., 2014), and it was suggested by Åsman et al. (2016) that these AGOs could mediate RNA regulation in a cleavage-independent way.

Small RNAs were observed to map to several Crinkle and Necrosis (CRN) and RxLR effectors of *Ph. infestans*, *Ph. parasitica* and *Phytophthora sojae* (Vetukuri et al., 2012; Jia et al., 2017; Wang et al., 2019). Since these two effector classes are known for their essential roles in overcoming plant immune responses (Anderson et al., 2015; Amaro et al., 2017; Liu et al., 2019), it is likely that RNA silencing has an important role in the regulation of key pathogenicity systems during the induction of *Phytophthora* diseases and evasion of detection by resistance proteins. Furthermore, sRNAs from oomycetes can occasionally be transferred directly to host organisms and carry out a role in virulence by “hijacking” the RNA silencing mechanism of their host, as was proven in *Hyaloperonospora arabidopsidis* infecting *Arabidopsis thaliana* (Dunker et al., 2020). Cross-kingdom and cross-species RNA silencing is not only observed in pathogens, but it is quickly being discovered or predicted in a number of biocontrol organisms. For example, *Beauveria bassiana* can use the milRNA bba-milR1 to attenuate the immune response of its host *Anopheles stephensi* (a mosquito vector of malaria parasites) (Cui et al., 2019), while many sRNAs of the mycoparasite *Clonostachys rosea*, another fungal species, were predicted to target virulence factors in *Botrytis cinerea* and *Fusarium graminearum* (Piombo et al., 2021, 2022). However, the potential for cross-species RNA silencing has never been assessed in biocontrol oomycetes to date.

The aim of this study was to analyze RNA silencing components and sRNA populations of oomycetes with diverse lifestyles, for which RNA silencing mechanisms have so far been unexplored, to investigate whether sRNAs are a major determinant of oomycete lifestyles, ranging from plant pathogenic to fungus and mosquito parasitic. The species to be analysed here are *L. giganteum*, a parasite of several mosquito species, the mycoparasites *Py. oligandrum* and *Py. periplocum*, and the *Phytophthora* plant pathogens *Ph. cactorum*, *Ph. colocasiae* and *Ph. plurivora*. *Lagenidium giganteum* has been used as a BCA against mosquitoes, while *Py. oligandrum* and *Py. periplocum* are used against plant pathogenic fungi and oomycetes (Paul, 1999; Bělonožníková et al., 2022). We also included the fungal biocontrol species *C. rosea* as an outgroup for comparisons. To achieve the aim we: (1) identified genes coding for DCLs, AGOs and RDRPs in the considered genomes, (2) performed sRNA sequencing of these species and analyzed the sRNA populations to ascertain differences in size, sequence bias and mapping between different sRNA populations, with emphasis on sRNAs mapping to known effector classes, (3) predicted milRNAs and milRNA targets, with a focus on putative cross-species targets with potential role in the biocontrol action of *L. giganteum*, *Py. oligandrum* and *Py. periplocum*. The results presented constitute an initial survey of how RNA silencing can affect the regulation of genes involved in pathogenesis and biocontrol in oomycetes, paving the way for practical applications in the field (Kalyandurg et al., 2021).

## Materials and methods

### Oomycete genomes and gene prediction

The genomes used in this project are *C. rosea* IK726 (GCA_902827195.1), *L. giganteum ARSEF 373* (GCA_002286825.1)*, Ph. cactorum* P414 (GCA_016864655.1)*, Ph. colocasiae* 7,290 (GCA_002288995), *Py. oligandrum* CBS 530.74 (Kushwaha et al., 2017a), *Py. periplocum* CBS 532.74 (Kushwaha et al., 2017b) and *Ph. plurivora* AV1007 (GCA_002247145.1). As gene predictions were not available for *L. giganteum*, *Ph. colocasiae, Ph. plurivora*, *Py. oligandrum* and *Py. periplocum*, genes were predicted using Augustus v3.4 (Stanke et al., 2006) and MAKER v3.01.1 (Campbell et al., 2014; Supplementary Files 1, 2). Species-specific parameters for Augustus were obtained by Augustus online training using available proteins from other oomycete species (Stanke and Morgenstern, 2005). Single copy genes present in all the considered species were identified with Orthofinder v2.5.2 (Emms and Kelly, 2019), concatenated and aligned with mafft v7.453 (Katoh and Standley, 2013) with the “-auto” option to select the best parameters. A phylogenetic tree was then obtained using iqtree2 v2.1.3 (Nguyen et al., 2015) with the option “-m MFP” to select the best model. The trees were visualized with FigTree v1.4.4 (Rambaut, 2018).

### Identification and phylogenetic analysis of RNA silencing proteins

The predicted proteomes of the considered species were annotated with InterProScan v5.46–81.0 (Jones et al., 2014). Proteins having both the ribonuclease III domain (IPR000999) and the Dicer dimerisation domain (IPR005034) were considered to be DCLs. Proteins having both the PAZ domain (IPR003100) and PIWI domain (IPR003165) were considered to be AGO proteins. Proteins having the eukaryotic type RDRP domain (IPR007855) were considered to be RDRPs.

Each category of proteins was aligned with mafft v7.453 (Katoh and Standley, 2013) with the “--maxiterate 1,000 --localpair” options, and a phylogenetic tree with 1,000 bootstraps was constructed using iqtree2 v2.1.3 (Nguyen et al., 2015) with the option “-m MFP” to select the best model. Both alignment and phylogenetic tree construction were run using only the conserved domains of interest (IPR000999, IPR005034, IPR003100, IPR003165, IPR007855). The trees were visualized with FigTree v1.4.4 (Rambaut, 2018) and they were rooted at *C. rosea*.

### Sample preparation, sRNA extraction and sequencing

*Ph. colocasiae*, *Ph. cactorum*, *Ph. plurivora*, *Py. oligandrum*, and *Py. periplocum* were individually cultured in liquid V8 juice media and *L. giganteum* on PYG liquid medium (Domnas et al., 1977) for 3 days at 20°C and collected by gravity filtration. The different culture samples were snap frozen in liquid nitrogen and kept at −70°C until RNA extraction. Total RNA was obtained using the Ambion mirVana miRNA isolation kit according to the manufacturer’s instructions (Invitrogen, Waltham, MA), and RNA quality was assessed using Agilent Bioanalyzer chips (Agilent Technologies, Santa Clara, CA). The sequencing was carried out at the SciLife sequencing facility in Stockholm using an Illumina HiSeq2500 sequencing platform in High Output mode, SR 1x50bp. FastQC v0.11.3 (Andrews, 2010) was used to examine the quality of raw reads.

### Analysis of sRNA sequences

Adapters, low quality and low complexity reads were removed with bbduk v38.86 (Bushnell, 2019) using the following options:

Bbduk.sh in = raw.fq out = clean.fq ref. = adapters.fa ktrim = r k = 23 mink = 11 hdist = 1 qtrim = r trimq = 10 maq = 10 minlen = 18 entropymask = f entropy = 0.5 entropywindow = 18 entropyk = 5.

The resulting reads were checked with FastQC v0.11.3 (Andrews, 2010). Afterwards, “reformat.sh” from the BBTools package v38.86 (Bushnell, 2019) was used to remove reads shorter than 18 bp or longer than 32 bp, and structural RNAs (rRNAs, tRNAs, snoRNAs and snRNAs) were removed using SortMerRNA v4.3.4 (Kopylova et al., 2012). The resulting reads were mapped to the respective genomes using bowtie v1.0.0 (Langmead, 2010) with the following options:

bowtie -S -p 10 -k 101 -v 1 -m 20 --best --strata bowtie_index reads.fq output.sam.

FeatureCounts v2.0.0 (Liao et al., 2014) was used to determine the number of reads mapped to various genomic features, using the following options:

featureCounts --fracOverlap 0.7 -O --fraction -g Parent -t genomic_feature_of_interest -a file.gff -o counts.txt BAMfiles.

### Functional annotation and detection of regions of interest

InterProScan v5.46.81.0 (Jones et al., 2014) was used to predict proteases. Diamond and HMMer were used on the dbCAN and dbCAN_sub databases through the dbCAN2 metaserver, and any gene predicted as a CAZyme by one of these programs was considered a CAZyme in subsequent analyses (Buchfink et al., 2015; Prakash et al., 2017; Zhang et al., 2018). Putative effectors were determined from these groups by predicting secretion using the procedures recommended previously (Gogleva et al., 2018). SignalP v4.0 (Petersen et al., 2011) was used to predict proteins possessing a signal peptide for secretion. Proteins with transmembrane domains were identified with TMHMM v2 (Krogh et al., 2001) and excluded from the analysis of effectors. TargetP v2 was then used to exclude proteins targeted to mitochondria (Almagro Armenteros et al., 2019) and PredGPI was used to remove proteins with a GPI anchor (Pierleoni et al., 2008). CRN and RxLR effectors were specifically predicted using effectR (Tabima and Grünwald, 2019).

Duplicated genes were determined with BLAST, comparing every proteome to itself with minimum identity of 85% and minimum query coverage of 90%. Antisense overlapping locations were determined with an *ad hoc* Python script using the Pandas module (McKinney, 2010), setting promoters to be 500 bp in length using promoter_extractor.^1^ When a promoter would have overlapped the exons of the previous gene on the same strand the promoter length was set so it commenced after the previous gene. The coordinates of introns and untranslated region (UTR) sequences (when available from gene prediction) were included in the gff files using, respectively, the programs “add_utrs_to_gff”^2^ and GenomeTools with the “-addintrons” option (Gremme et al., 2013).

Transposons and repeat sequences were predicted with RepeatModeler v.2.0.1 (Flynn et al., 2020) and coordinates were obtained through RepeatMasker v. 4.1.1 (Smit et al., 2015). The data was analyzed using the Python module Pandas (McKinney, 2010) and visualization was carried out with the Python module Seaborn (Bisong, 2019).

### milRNA analysis

milRNAs were predicted with mirdeep2 v.2.0.0.7 (Friedländer et al., 2012) and they were retained only if they were present in at least 10 counts on average in every sample of the considered species. Targets were predicted using TargetFinder and psRNAtarget, as well as TAPIR and psRobot through the online sRNAtoolbox (Bo and Wang, 2005; Bonnet et al., 2010; Wu et al., 2012; Rueda et al., 2015; Dai et al., 2018). Predictions supported by at least two programs were considered in further analysis. Prediction of candidate transboundary milRNA targets was carried out using the same programs for milRNAs of *Py. oligandrum* and *Py. periplocum*. Available transcripts of *Ph. infestans* (GCF_000142945.1) and *B. cinerea* (GCF_000143535.2) were used as putative targets, and only predictions supported by all four software packages were considered. Prediction of candidate transboundary targets for milRNAs of *L. giganteum* was performed using the animal-dedicated tools Miranda, PITA and TargetScan, used online through the sRNAtoolbox (Rueda et al., 2015). The targets were 3' UTR regions of insect host *Aedes aegypti* (GCF_002204515.2), where coordinates were derived from the gff file using “add_utrs_to_gff” (see Footnote 2). Only predictions confirmed by all three software packages were considered.

## Results

### Identification and phylogenetic analysis of DCLs, AGOs, RDRPs

We performed domain prediction in the species of interest to identify the components of the RNA silencing mechanism. All of the oomycete genomes analysed contained a single gene encoding a DCL, while the number of AGO and RDRP coding genes was variable (Table 1). The number of AGO coding genes was lowest in *Py. periplocum* (two genes), while *Ph. plurivora* had six AGO coding genes. One AGO coding gene, similar to *Ago1* and/or *Ago2* from *Ph. infestans* (clade 1) was identified in the analysed species, with the exception of two genes in *L. giganteum*. All other AGOs were similar to *Ago3/4/5* from *Ph. infestans* (clade 2). A single RDRP gene was predicted in the species analysed, with the exception of two and five genes in *Py. periplocum* and *L. giganteum*, respectively. Some of the predicted DCL proteins show a P-loop NTPase fold (IPR027417) or a DEAD box helicase domain (IPR011545), in addition to the Dicer dimerization domain (IPR005034) and the ribonuclease III domain (IPR000999). Similarly, all AGOs have a combination of protein argonaute N-terminal domain (IPR032474), Argonaute linker 1 and linker 2 domains (IPR014811, IPR032472) and Argonaute mid domain (IPR032473), in addition to the PAZ (IPR003100) and PIWI (IPR003165) domains. All the domains detected in the considered proteins are listed in Supplementary Table 1.

**Table 1:** Copy number of genes coding for DCLs, AGOs and RDRPs in the species considered in this study.

Enzyme class	*C. rosea*	*Py. oligandrum*	*Py. periplocum*	*Ph. cactorum*	*Ph. colocasiae*	*Ph. plurivora*	*L. giganteum*
Dicers	2	1	1	1	1	1	1
Clade 1 Argonautes	1	1	1	1	1	1	2
Clade 2 Argonautes	1	2	1	3	4	5	2
RDRPs	3	1	2	1	1	1	5

**Figure 1:**
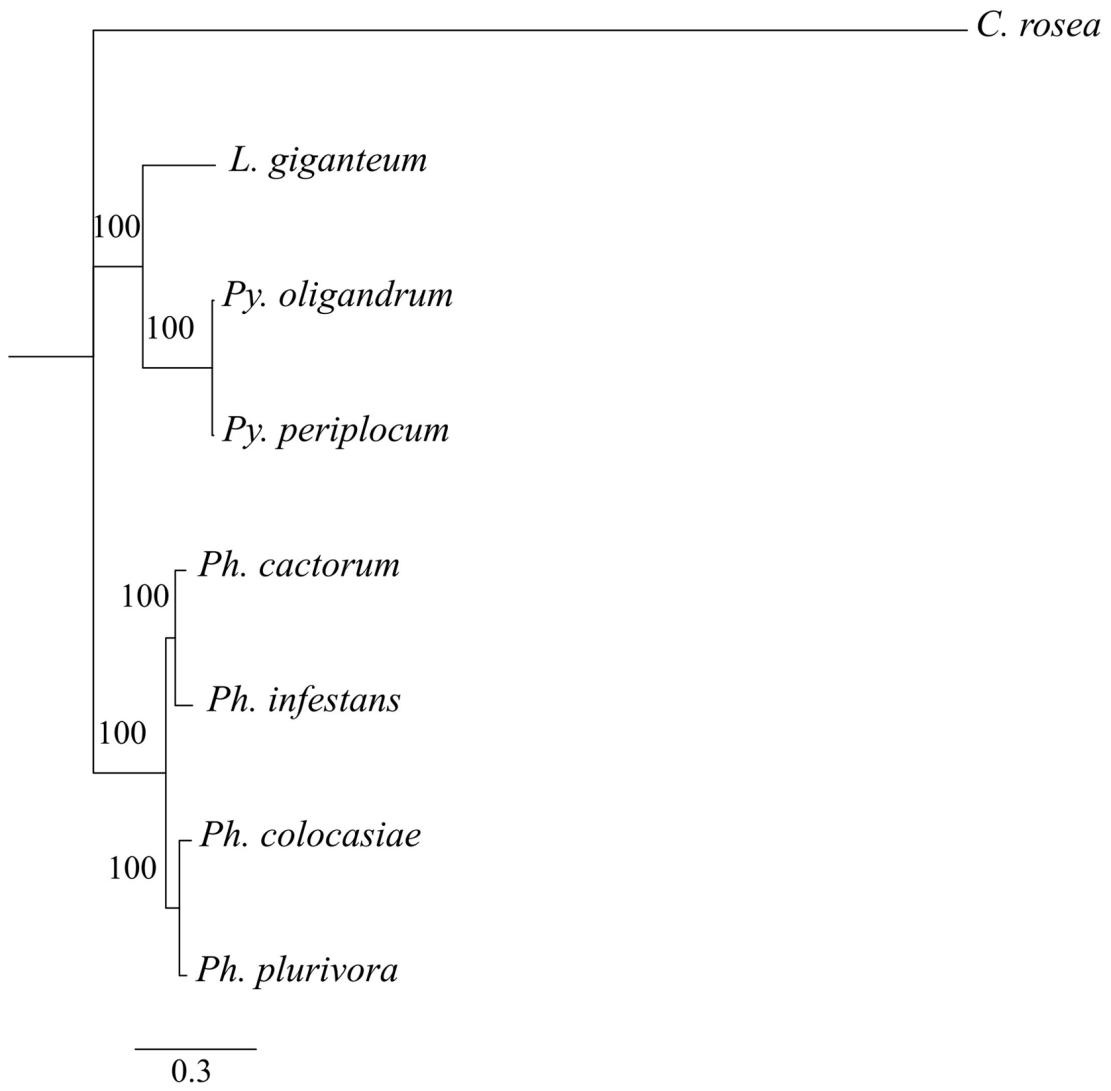
Phylogenetic tree illustrating phylogenetic relationships of the species of interest, based on the concatenation of all single-copy proteins present in every species. Rapid bootstrap support (≥60%) values from 100,000 iterations are associated with nodes.

**Figure 2:**
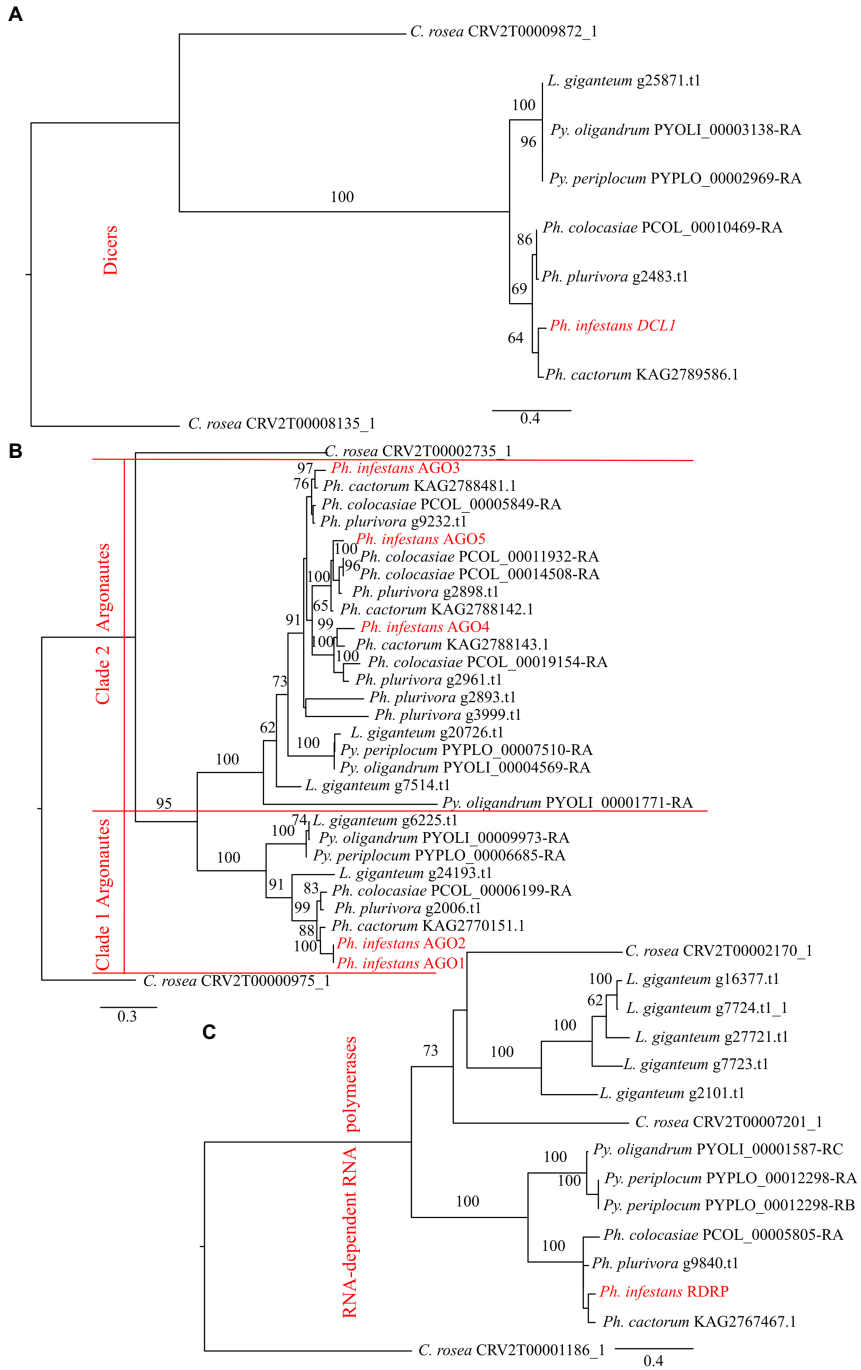
Phylogenetic tree illustrating phylogenetic relationships of: DCL proteins **(A)**, AGO proteins **(B)** and RDRP proteins **(C)**. The two AGO clades are separated by red lines. Bootstrap support (≥60%) values from 1,000 iterations are associated with nodes.

Phylogenetic analysis showed that DCLs clustered according to the expected evolutionary relationships between the species (Figure 1), with both *Pythium* spp. clustering together and separately from the *Phytophthora* spp. Both of these groups clustered separately from *C. rosea*, while *L. giganteum* clustered with the *Pythium* spp. (Figure 2A). AGOs clustered in two main clades according to their similarity to AGO1/2 or AGO 3/4/5 of *Ph. infestans*, again with a clear separation from *C. rosea.* In each of these two clusters, *Pythium* AGOs were separate to those from *Phytophthora*, and *L. giganteum* proteins were closer to those from *Pythium* (Figure 2B). Contrary to the DCL and AGO phylogenies, *L. giganteum* RDRP proteins clustered separately from both *Pythium* and *Phytophthora* RDRPs, and were closer to those from *C. rosea* (Figure 2C).

A second class of DCL proteins, similar to *Ph. infestans* DCL2, was also detected in the considered oomycetes. This class has two RNaseIII domains but no Dicer dimerization domain and it is more similar to the Drosha protein. All the considered *Pythium* spp. and *Phytophthora* spp. had one gene belonging to this class in their genomes. *Pythium* DCL2 proteins clustered separately to those from *Phytophthora*. *L. giganteum* was the only exception as it had two genes coding for DCL2 proteins; one was similar to *Phytophthora* DCL2 and the other was separate to the DCL proteins of the other oomycetes (Supplementary Figure 1).

### sRNA length and 5′ base preference

Sequencing of sRNAs produced between 7.9 and 60 million high-quality reads for each sample. After removal of structural RNAs (ribosomal RNAs, transfer RNAs, small nuclear RNAs and small nucleolar RNAs), as well as reads shorter than 18 or longer than 32 nt, the final numbers of sequences ranged between 6.6 million and 27.1 million reads (Supplementary Table 2). *L. giganteum* yielded the highest proportion of usable reads (between 70 and 83% of the reads were kept when filtering for size and non-structural RNAs), while *Py. periplocum* had the lowest proportion (between 13 and 14% of total reads were usable).

**Figure 3:**
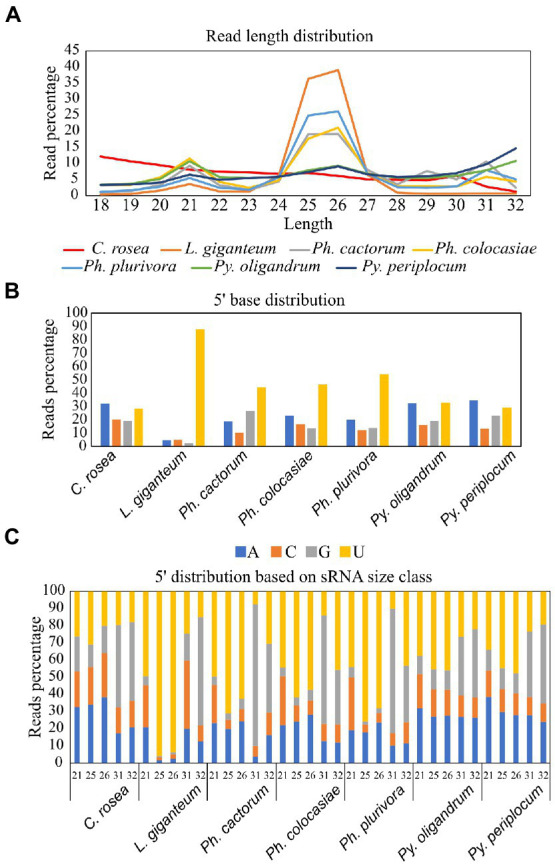
Characteristics of sRNAs identified in *Pythium* spp., *Phytophthora* spp., *Clonostachys rosea* and *Lagenidium giganteum.* **(A)** Read length distribution. **(B)** 5′ base distribution. **(C)** 5′ base distribution based on sRNA size class.

Average length distribution of sRNAs revealed that all oomycete species analysed exhibited clear peaks at 21 nt and 25/26 nt, but with differences in the level of these peaks. The *Phytophthora* spp. analysed had a further distinct sRNA size peak at 31 nt. *L. giganteum* had an overall size distribution and abundance pattern that appeared most similar to the *Phytophthoras*. 25/26 nt sRNAs represented 40% of reads in *Ph. cactorum* and *Ph. colocasiae*, 50% in *Ph. plurivora* and 75% in *L. giganteum*. *Pythium* spp. had smaller peaks for sRNAs of 21, 26 and 32 nt, and none of them amounted to more than 15% of the reads (Figure 3A).

In *C. rosea* and *Pythium* spp. samples around 30% of sRNAs had an adenine or uracil at the 5′ end. Approximately 20% of *C. rosea* sRNAs had a 5′ guanine and cytosine. In the *Pythium* spp., approximately 20% of sRNAs had a 5′ guanine, while approximately 15% had a 5′ cytosine. In comparison, in all *Phytophthora* spp. there was a clear preference for uracil at the 5′ end of sRNAs, up to 54% in *Ph. plurivora*. This phenomenon was even more pronounced in *L. giganteum*, in which 88% of sRNAs had a 5′ uracil (Figure 3B). In all oomycete species assessed here, the prevalence of uracil at the 5′ end increased for sRNAs of 25/26 nt, with the most extreme example being approximately 95% of sRNAs in this size class in *L. giganteum* (Figure 3C). While guanine was the least preferred 5′ base for 21 nt and 25/26 nt sRNAs, especially in *Phytophthora* and *L. giganteum*, it was typically the most common 5′ base for longer sRNAs of 31/32 nt, with the exception of *L. giganteum* which had cytosine as the most common base at the 5′ end of 31 nt sRNAs. In the two *Pythium* spp., the 21 nt sRNAs exhibited similar preference for a 5′ adenine or uracil (30–40%), with lesser proportions of cytosine and guanine (10–20%). In contrast, 21 nt sRNAs from *Phytophthora* and *L. giganteum* exhibited a preference for 5′ uracil (45–50%), a strong bias against guanine (less than 10%), and a higher proportion of 5′ cytosine (20–30%) (Figure 3C).

### Analysis of sRNA mapping to duplicated genes, transposons and antisense overlapping regions

**Figure 4:**
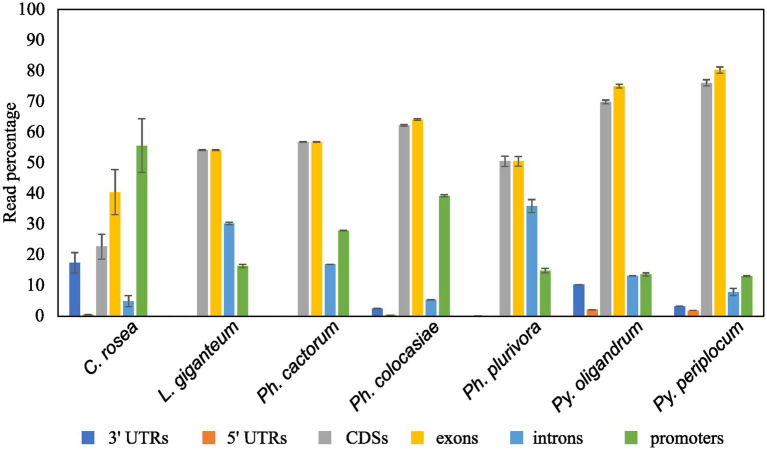
Proportion of sRNA reads mapped to different genic locations of the respective species. Error bars indicate the standard deviation between the replicates. The percentage is calculated on the total number of reads mapping to genes.

**Figure 5:**
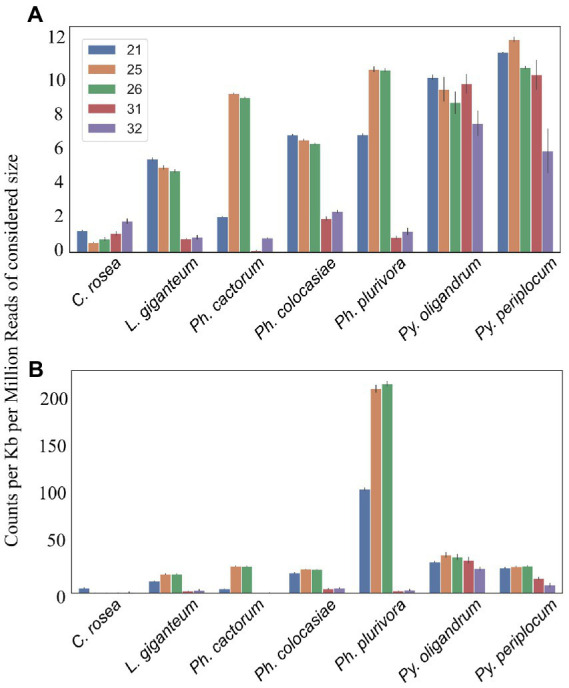
Number of reads mapping to transcripts. Data normalized according to total kb extent of the transcriptome in the genome and to millions of reads of the specific sRNA size for the sample. Error bars indicate the standard deviation. **(A)** All transcripts. **(B)** Duplicated transcripts.

Mapping of sRNAs to the respective genomes revealed that a majority of sRNAs originated from intergenic regions. This portion of the reads was highest (more than 80%) in *C. rosea*, while it was lowest in *Py. oligandrum* and *Py. periplocum* amounting to less than 50% of the total sRNA reads. Among the reads mapping to genetic features (Figure 4), sRNA reads from oomycetes mapped more frequently to exons than to promoters or introns, while in the ascomycete *C. rosea* a higher proportion of sRNAs mapped to promoters. Most of the reads assigned to exons were assigned to CDS regions. UTRs could only be predicted for *C. rosea*, *Ph. colocasiae*, *Py. oligandrum* and *Py. periplocum*, and in every case the 3’ UTR had more sRNAs mapped to it than the 5’ UTR. *Ph. cactorum* and *Ph. colocasiae* had similar mapping statistics, with exons/CDSs as the source of most sRNAs, followed by promoters and then introns. *Ph. plurivora*, however, had more sRNAs mapping to introns than to promoters, and the same was true for *L. giganteum*. In the two *Pythium* spp. *Py. oligandrum* had similar numbers of sRNAs mapping to promoters and introns, while in *Py. periplocum* promoters were the origin of almost double the sRNAs mapping to introns. Even after normalizing according to the number of reads of each length, 25 and 26 nt sRNAs were still the most likely classes mapping to genes in *Ph. cactorum* and *Ph. plurivora,* while a peak at 21 nt was also present in *L. giganteum* and *Ph. colocasiae* and peaks at 21 nt and 31 nt were present in the *Pythium* spp. (Figure 5A).

In the analysed oomycete species, a higher number of sRNAs mapped to duplicated genes, ranging from 25 to 50 counts per kb of duplicated genes per million reads of the 21, 25 and 26 nt sRNAs compared to 2–12 sRNA counts when all genes were considered (Figure 5B). The sRNA size classes most likely to be mapped to duplicated genes were the same which mapped to single genes. The number of 25 nt and 26 nt sRNAs mapping to duplicated transcripts was particularly high in *Ph. plurivora,* with peaks of over 200 counts per kb of duplicated genes per million reads (Figure 5B). GO term enrichment analysis of duplicated genes from oomycetes species used in this study showed enrichment of the GO term “zinc ion binding” (GO:0008270). In addition, the GO term “FMN binding” (GO:0010181) was enriched in the duplicated genes of both *Pythium* spp., but not in the *Phytophthora* spp. (Supplementary Table 3).

**Figure 6:**
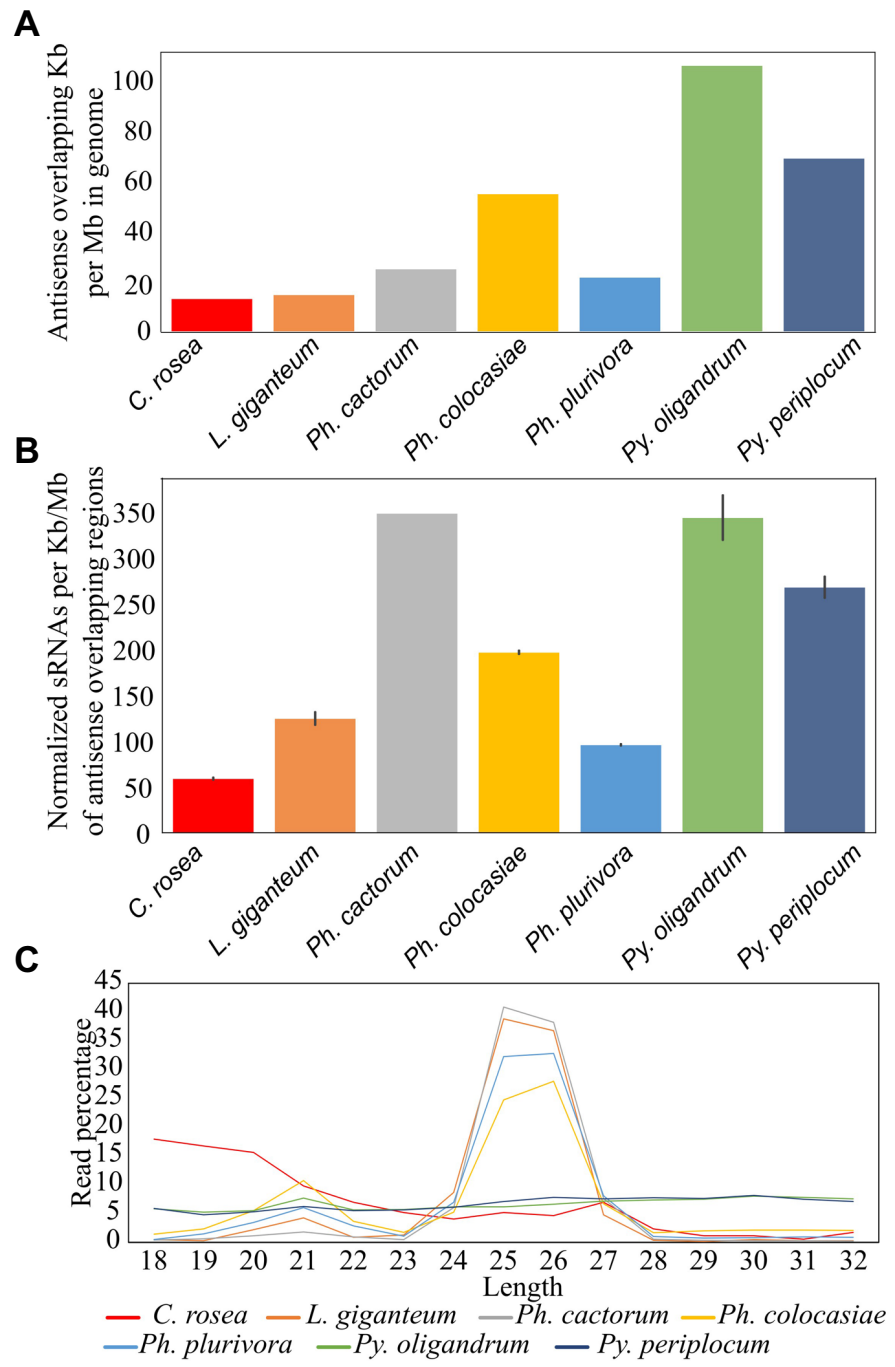
Mapping of sRNAs to antisense overlapping regions. **(A)** Extent of antisense overlapping regions in the analyzed species. **(B)** Number of sRNA reads mapping to antisense overlapping regions. Data normalized according to millions of reads per sample, length of the genomes and length of the antisense overlapping regions. Error bars indicate the standard deviation. **(C)** Length distribution of sRNAs mapping to antisense overlapping regions.

Among the species analysed, *Py. oligandrum* and *Py. periplocum* had the highest amount of antisense overlapping regions (79 kb - 100 kb per Mb), followed by *Ph. colocasiae* at 55 kb per Mb (Figure 6A). However, after normalization, according to the length of these regions, *Ph. cactorum* and *Py. oligandrum* sRNAs showed higher mapping (350 sRNAs per kb) to antisense overlapping regions per million mapped reads, followed by *Py. periplocum* and *Ph. colocasiae* with 279 and 190, respectively. In *C. rosea*, *L. giganteum* and *Ph. plurivora*, between 130 and 50 sRNAs were mapped per kb of antisense overlapping region (Figure 6B).

The most frequent length for oomycete sRNAs mapping to antisense overlapping regions was 25–26 nt in *L. giganteum* and the *Phytophthora* spp., while these peaks were not present in the *Pythium* spp. All oomycete species analysed also had a second peak at 21 nt, while the length distribution of *C. rosea* reads was very different from that of the oomycetes and presented no distinct peaks, even though the most common length for sRNAs mapping to antisense overlapping regions was 18 bp (Figure 6C). The 31 nt peak observed in the total sRNA population was absent in all species analysed when sRNA were mapped to antisense overlapping regions.

**Figure 7:**
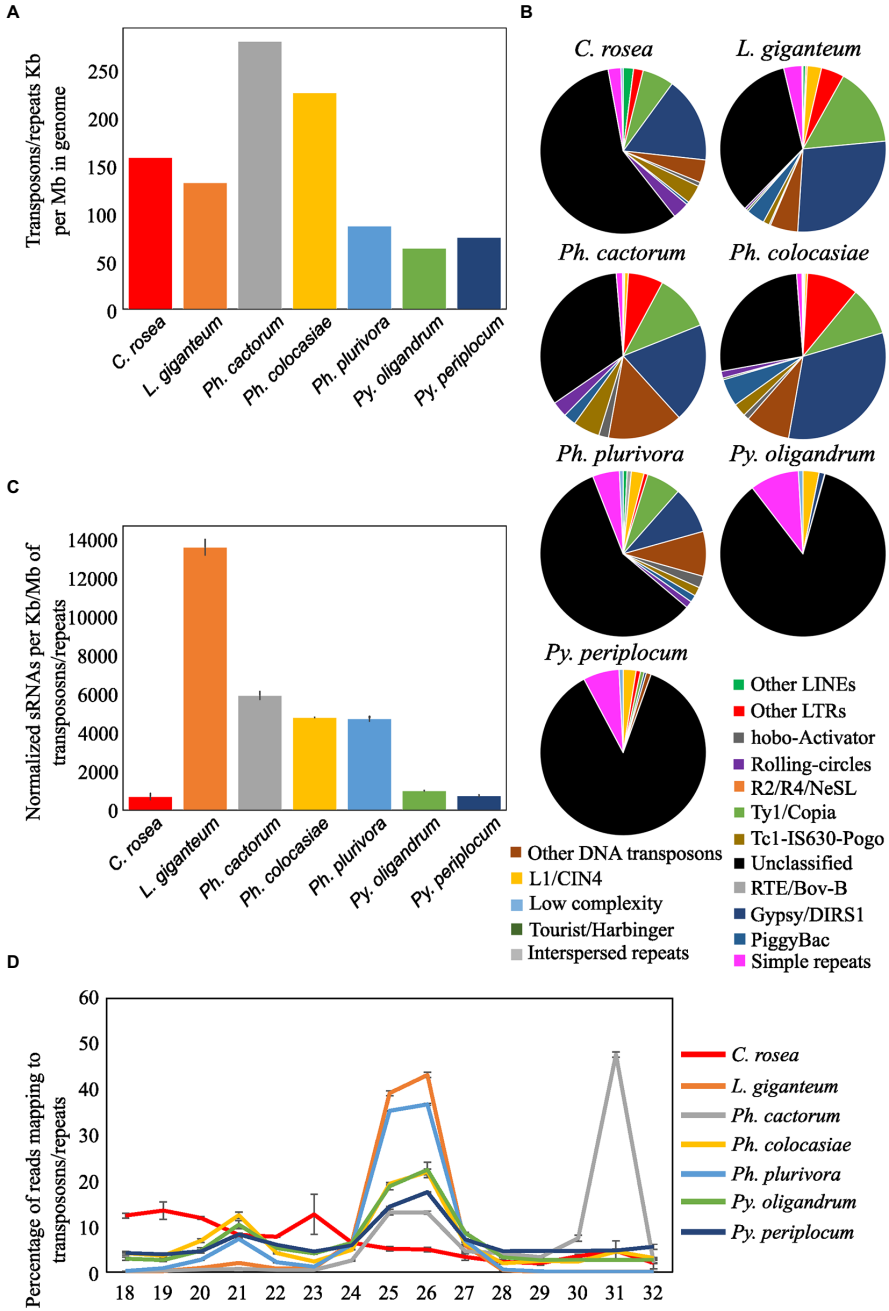
Identification of transposons and repeated sequences with RepeatModeler. **(A)** Extent of transposons and repeated sequences in the analyzed species. **(B)** Families of transposons identified in the genomes of the species of interest. **(C)** Number of sRNA reads mapping to transposons and repeated sequences. Data normalized according to millions of reads per sample, length of the genomes and length of the repetitive regions. Error bars indicate the standard deviation. **(D)** Length distribution of sRNA mapping to transposons and repeated sequences.

Transposons and repetitive regions were more frequent in *Ph. cactorum* (around 279 kb repeat/transposon per Mb of genome), followed by *Ph. colocasiae* at more than 200 kb/Mb and then *C. rosea*, *L. giganteum*, *Ph. plurivora*, *Py. periplocum*, and *Py. oligandrum*, with the last three having between 50 and 100 kb/Mb (Figure 7A). The most abundant repeat/transposon in *Phytophthora* spp. were LTRs, in particular those of classes Copia and Gypsy, followed by DNA transposons. In *Pythium* spp., on the other hand, around 85% of transposons and repeated sequences were unclassified, with the most common recognized class being simple repeats (Figure 7B). The extent of repeated regions was not a good indicator of the number of sRNAs mapping to them. In particular, *L*. *giganteum* was by far the species with the highest number of sRNA reads mapping to repeats/transposons, reaching almost 14,000 reads per kb of repeats per Mb of genome, normalized also according to sequencing depth. The analysed *Phytophthora* spp. ranged between 4,500 and 6,000 sRNAs per kb/Mb of repeats, with *Ph. plurivora* reaching a similar value to the other two *Phytophthora* spp. despite having much less of its genome composed of repeat/transposon sequences. *Pythium* spp. and *C. rosea*, however, had less than 1,000 sRNAs per kb/Mb of repeat/transposon. (Figure 7C). Most of the sRNAs mapping to transposons and repetitive regions of oomycetes had a length of 25–26 nt, with the exception of *Ph. cactorum* which had the major peak at 31 nt. Moreover, *Ph. colocasiae*, *Ph. plurivora* and both *Pythium* spp. also had a peak at 21 nt. *C. rosea* had a different distribution from the oomycetes and had peaks at 18–20 nt and 23 nt (Figure 7D).

### CRN and RxLR effectors

**Figure 8:**
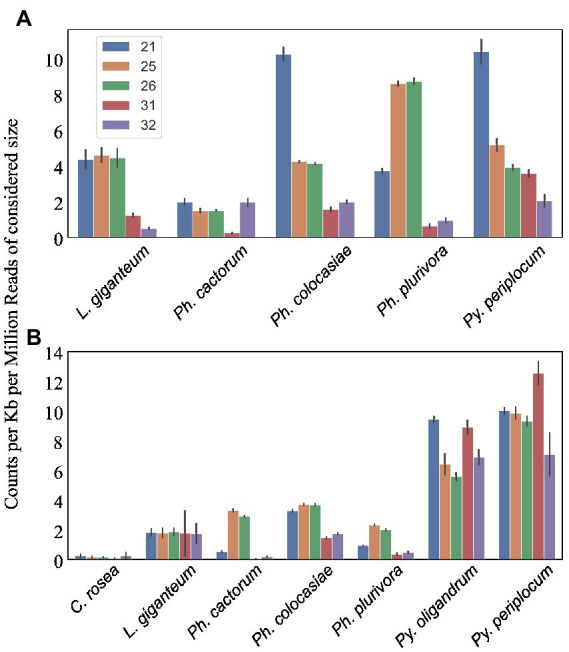
Number of sRNA reads mapping to putative effectors detected in the species of interest. Data normalized according to total kb extent of the respective effector area in the genome and to millions of reads of the specific sRNA size for the sample. Error bars indicate the standard deviation. **(A)** CRNs. **(B)** RxLRs.

We predicted CRNs and RxLR genes in the genomes of interest, as these are two important classes of effectors in oomycete plant pathogens (Anderson et al., 2015; Amaro et al., 2017). Genes encoding CRNs were detected in every oomycete species analysed except *Py. oligandrum*, with the highest number identified in *Ph. cactorum* (230 putative CRN coding genes), while 85 genes coding for CRNs were detected in *L. giganteum* (Table 2). After normalizing according to the length of regions covered by these genes, the sizes of sRNAs mapping to predicted CRNs appeared to be species-specific, with 21 nt sRNAs more likely to map to these transcripts in *Ph. colocasiae* and *Py. periplocum,* while 25 nt and 26 nt sRNAs were more common in *Ph. plurivora,* and 21, 25 and 25 nt sRNAs were equally likely to target CRNs in *L. giganteum* and *Ph. cactorum* (Figure 8A).

**Table 2:** Number of putative secreted proteins, including effectors, predicted in fungal and oomycete species analyzed in this study.

Effector category	*C. rosea*	*Py. oligandrum*	*Py. periplocum*	*Ph. cactorum*	*Ph. colocasiae*	*Ph. plurivora*	*L. giganteum*
Proteins	21,246	17,141	14,586	29,905	23,815	11,749	28,904
Secretome	1,504	811	656	1,358	885	773	1,449
Secreted CAZymes	331	140	119	276	177	223	189
Secreted proteases	272	168	118	136	78	43	386
Other secreted proteins	1,045	587	476	818	542	423	1,066
Secreted RxLRs	0	0	0	177	126	75	0
Secreted CRNs	0	0	2	19	2	15	1
RxLRs	25	20	27	373	346	195	25
CRNs	0	0	115	230	190	129	85

Genes coding for proteins with putative RxLR motifs, on the other hand, were predicted in every species, again with the highest number (373 genes) detected in *Ph. cactorum*. In *Phytophthora* spp., the amount of 25–26 nt sRNAs mapping to these effectors was proportionally higher than for other sizes of sRNAs (Figure 8B), although in *Ph. colocasiae* 21 nt sRNAs were almost as likely to target RxLRs as 25 and 26 nt sRNAs (Figure 8B). The *Pythium* spp. had a low number of putative RxLRs (20 to 27), and they were on average more targeted by sRNAs than those in *Phytophthora*. These genes encoded proteins with the typical RxLR-EER motifs, but lacked predicted signal peptides, had no homology with any previously described RxLR effectors (Tabima and Grünwald, 2019), and it is likely they perform other functions in the *Pythium* spp., as the RxLR motif can sometimes occur by chance. This is likely the case also for the few RxLR genes identified in *C. rosea* and *L. giganteum*.

**Figure 9:**
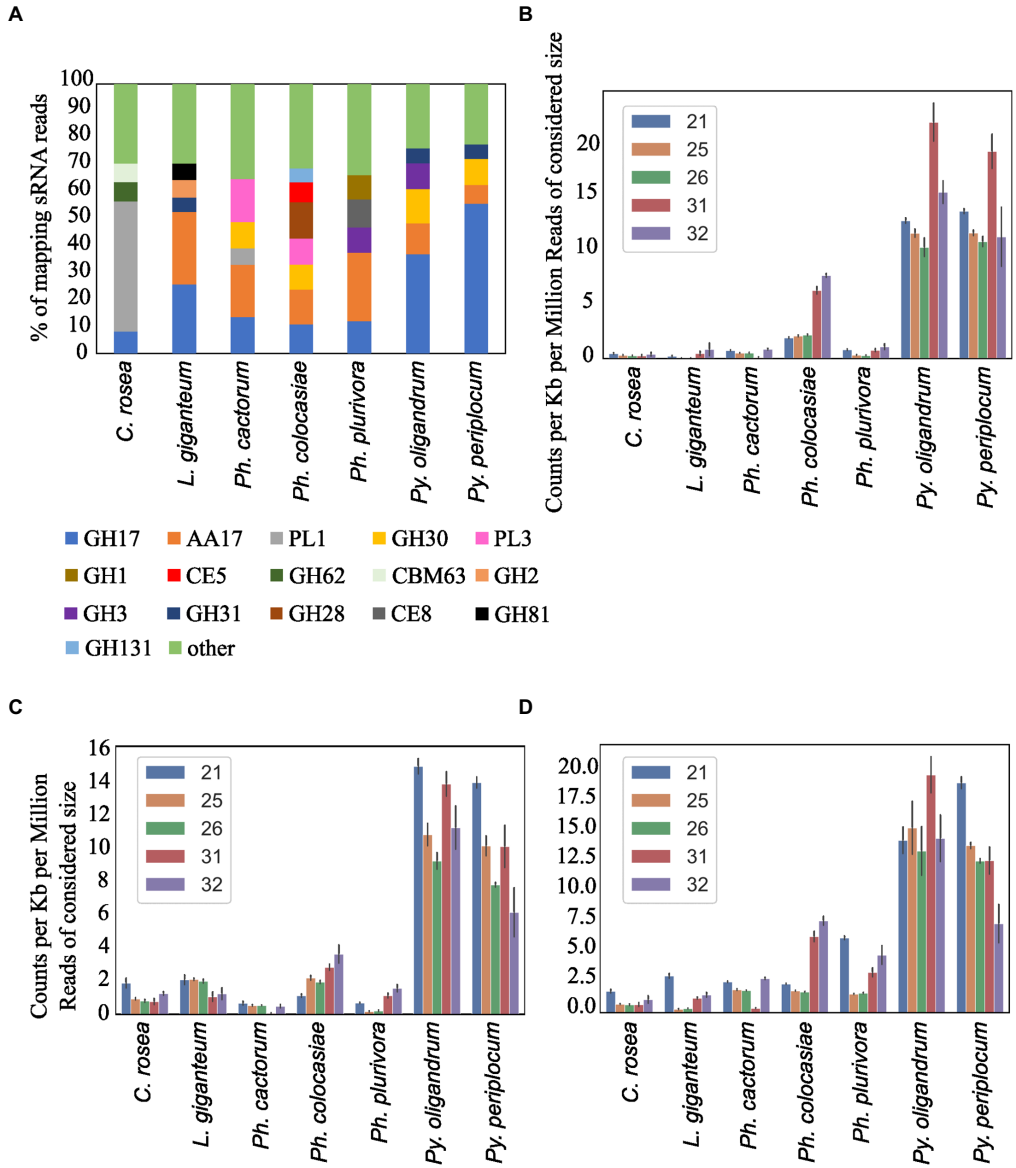
Distribution of the sRNA reads mapping to secreted CAZyme families in the species of interest **(A)**. Number of reads mapping to secreted CAZymes **(B)**, proteases **(C)** and secreted proteins not predicted to be CAZymes, proteases, CRNs or RxLRs **(D)**. Data normalized according to total kb extent of the respective gene area in the genome and to millions of reads of the specific sRNA size for the sample. Error bars indicate the standard deviation.

Secreted CAZymes often have roles in interactions with the host organism. *Ph. cactorum* and *Ph. plurivora* had the highest number of these proteins (276 and 223, respectively), while *Py. oligandrum* and *Py. periplocum* had 140 and 119, respectively. Considering the sRNAs that mapped to genes encoding secreted CAZymes, 56% of *Py. periplocum* sRNAs mapped to GH17 (1,3-β-glucosidases), which is considerably higher compared to *Phytophthora* spp. sRNAs (12% on average). In *Phytophthora* spp., a higher proportion of sRNA reads (19%) mapped to AA17 (copper-dependent lytic polysaccharide monooxygenases), followed by GH17. These two classes were the origin of most sRNAs even in *L. giganteum*. Interestingly, in *Ph. colocasiae* and *Ph. cactorum* more than 10% of sRNAs mapping to secreted CAZymes mapped to GH30 (xylanases and glucosidases) and PL3 (pectate lyases), while these were almost absent in *Ph. plurivora*, which had instead more sRNAs mapping to GH3 (glucosidases, xylosidases and alpha-L-arabinofuranosidases) and CE8 (pectin methylesterases) CAZymes (Figure 9A). Both *Pythium* spp. had proportionally more sRNAs of all sizes mapping to genes encoding secreted CAZymes, when compared with the *Phytophthora* spp. (Figure 9B). The largest proportion of reads mapping to secreted CAZymes were 31 nt length in both *Pythium* spp., and 32 nt sRNAs more commonly mapped to CAZyme coding genes than 21, 25 or 26 nt sRNAs in *Py. oligandrum*. In the *Phytophthora* spp., *Ph. colocasiae* had the highest number of sRNAs mapping to genes encoding secreted CAZymes, with a predominance of 31 nt and 32 nt sRNAs, while all size classes except 31 nt were equally present in *Ph. cactorum*. In *Ph. plurivora*, 21, 31 and 32 nt sRNAs were present in similar quantities. A low number of reads mapped to secreted CAZymes in *C. rosea* and *L. giganteum* compared to *Pythium* or *Phytophthora* spp.

Among the oomycete species analysed, *L. giganteum* had the highest number of secreted proteases (386), followed by *Py. oligandrum* with 168, while the species with the lowest number was *Ph. plurivora* with 43. As with CAZymes, *Pythium* spp. had proportionally more sRNAs mapping to secreted proteases, when compared with the other species (Figure 9C). Secreted proteases were associated mostly with 21 nt sRNAs in *Py. periplocum* and 21 and 31 nt sRNAs in *Py. oligandrum.* 31 nt and 32 nt sRNAs were more prevalent in *Ph. colocasiae* and *Ph. plurivora,* while 21, 25, 26 and 32 nt sRNAs were equally likely to map to a secreted protease gene in *Ph. cactorum.* 21, 25 and 26 nt sRNAs were the most prevalent size classes mapping to genes coding for secreted proteases in *L. giganteum*.

Other secreted protein coding genes were also the origin of more sRNAs in *Pythium* spp., especially 31 nt sRNAs in *Py. oligandrum* and 21 nt sRNAs in *Py. periplocum* (Figure 9D). sRNAs of 31 nt and 32 nt were proportionally more abundant in *Ph. colocasiae*, while 21 nt sRNAs were prevalent in *Ph. plurivora* and *L. giganteum,* and 21 nt and 31 nt sizes were prevalent in *Ph. cactorum.*

### Identification of milRNAs and their putative gene targets at endogenous and cross-species level

milRNA prediction resulted in the identification of 38 putative milRNAs from the insect parasitic oomycete *L. giganteum*, which is a markedly higher number compared to the number of milRNAs identified in mycoparasitic *Py. oligandrum* (five milRNAs) and *Py. periplocum* (three milRNAs). Among the *Phytophthora s*pp. a higher number of milRNAs were predicted in the taro leaf blight pathogen *Ph. colocasiae* (13 milRNAs) compared to two and three milRNAs in *Ph. cactorum* and *Ph. plurivora,* respectively. These milRNAs were novel and unique to the respective species with the exception of pol_mir_1-ppe_mir_1, which was present in both *Py. periplocum* and *Py. oligandrum*. The precursors of the majority of milRNAs were located in intergenic regions (Supplementary Table 4). Additional information on the predicted milRNAs is presented in Supplementary Table 4.

milRNAs normally perform their regulating action by inducing the degradation of target transcripts. Between two and 25 endogenous milRNA targets were predicted for each species (Table 3; Supplementary Table 5). The detected milRNAs were predicted to control a variety of genes, including a dynein protein of *Ph. colocasiae* (PCOL_00008544-RA), a CRN effector of *L. giganteum* (g26638.t1), an RxLR effector of *Ph. plurivora* (g9339.t1) and one of *Ph. cactorum* (Pcac1_g11221.t1). This last transcript shows high similarity (qcov >90% and ID >85%) to transcript U17742 identified in *Ph. cactorum* strain 10,300, upregulated in germinating cysts with germ tubes compared to mycelium and zoospores (Chen et al., 2018).

**Table 3:** Targets of interest predicted for the detected milRNAs.

Effector category	Producing species	Target gene	Function
Endogenous targets			
Lgi_mir_15	*L. giganteum*	g26638.t1	CRN effector
Pca_mir_1	*Ph. cactorum*	KAG2778520.1	RxLR effector
Pco_mir_12	*Ph. colocasiae*	PCOL_00008544-RA	Dynein
Ppl_mir_1	*Ph. plurivora*	g9339.t1	RxLR effector
Transboundary targets in *Ph. infestans* and *B. cinerea*			
Ppe_mir_2	*Py. periplocum*	PITG_00939 (*Ph. infestans*)	Endoribonuclease L-PSP
Ppe_mir_2	*Py. periplocum*	PITG_03209 (*Ph. infestans*)	Hypothetical protein, oomycete specific
Ppe_mir_2	*Py. periplocum*	PITG_13437 (*Ph. infestans*)	Metalloprotease family M16C
Ppe_mir_1	*Py. periplocum*	BCIN_07g03380 (*B. cinerea*)	GTPase activating protein
Ppe_mir_2	*Py. periplocum*	BCIN_14g01020 (*B. cinerea*)	Transmembrane alpha-helix domain-containing protein
Ppe_mir_2	*Py. periplocum*	Bccch1 (*B. cinerea*)	Calcium channel
Ppe_mir_2	*Py. periplocum*	BCIN_08g04100 (*B. cinerea*)	Membrane protein
Ppe_mir_2	*Py. periplocum*	BCIN_04g03280 (*B. cinerea*)	Homeobox and C2H2 transcription protein

Some milRNAs are known to be exported to interacting organisms and perform their regulation activity in host cells (Wang et al., 2016; Cui et al., 2019), therefore we predicted putative transboundary targets for our species of interest. MilRNA Ppe_mir_2 of *Py. periplocum* was predicted to target three genes of *Phytophthora infestans* (PITG_00939, PITG_03209, PITG_13437), encoding a putative diphthine-ammonia ligase, an oomycete-specifi c conserved hypothetical protein, and a metalloprotease, respectively, suggesting the potential for cross-species RNA silencing regulation in the biocontrol action of *Py. periplocum*. The same milRNA also had four putative gene targets in *B. cinerea* (Supplementary Table 5), consisting of two membrane proteins, one protein with a zinc finger C2H2-type domain (BCIN_04g03280) and Bccch1, a calcium channel involved in vegetative growth in conditions of low extracellular calcium (Harren and Tudzynski, 2013). These targets were copredicted for the same milRNA by four separate prediction programs, and no endogenous target was similarly copredicted for these *Py. periplocum* milRNAs. However, eight endogenous targets were copredicted by three programs for ppe_mir_2 (Supplementary Table 5). MilRNA Ppe_mir_1, on the other hand, had no endogenous gene targets co-predicted by three or four programs, but one putative transboundary gene target in *B. cinerea,* a putative GTPase activating protein (BCIN_07g03380) copredicted as a target by all four of the programs used (Supplementary Table 5). The same type of co-prediction was performed for milRNAs of *L. giganteum* and targets of *Aedes aegypti*, resulting in 5227 transcripts identified as putative targets.

## Discussion

This study into RNA silencing components in the oomycetes represents the broadest overview to date and reveals commonalities and some specific differences among this group of filamentous microbes. All oomycetes analysed here had DCLs, AGOs and RDRPs in their predicted proteomes.

The first step in RNA silencing involves cleavage of long dsRNA substrates into sRNAs by DCL RNaseIII proteins. Each oomycete analysed here had one DCL protein. In a previous study, a second, more divergent DCL containing two RNaseIII domains was identified from *Ph. infestans*, *Ph. sojae* and *Ph. ramorum* (Fahlgren et al., 2013). However, BLASTP similarity searches with this protein in this study revealed that it is most similar to Drosha proteins. Previous studies with *Ph. infestans* showed that DCL1 is associated with the generation of 21 nt sRNAs (Vetukuri et al., 2012). While DCL2 may be more similar to Drosha proteins, the presence of two RNaseIII domains might suggest its involvement in generation of longer sRNAs such as the 25/26 nt class. However, all the oomycete species considered in this study had a homolog of *Ph. infestans* DCL2 (with the exception of *L. giganteum*, which had two), irrespective of the differences detected in sRNA length distribution. Linking oomycete DCL2 with a specific size of sRNA remains to be determined experimentally.

Oomycete AGOs are divided into two clades (Vetukuri et al., 2011; Fahlgren et al., 2013; Åsman et al., 2016; Jia et al., 2017). Clade 1 comprises closely related proteins that have the four catalytic amino acids in their PIWI domain, which allows them to mediate target cleavage. Clade 2 contains a more diverse grouping of AGO proteins and does not have this group of catalytic amino acids. Instead, clade 2 AGOs are suggested to mediate mRNA regulation in a cleavage-independent way (Åsman et al., 2016). Clade 1 is furthermore associated mostly with 21 nt sRNAs and a preference for cytosine at the 5′ end, as well as with a post-transcriptional regulation role, while clade 2 is associated with 25/26 nt sRNAs, a preference for uracil at the 5′ end, and a regulation role at the transcriptional level (Vetukuri et al., 2012; Fahlgren et al., 2013; Åsman et al., 2016; Jia et al., 2017). In plants like *A. thaliana* there is also a similar separation, with AGO3 and AGO4 binding mostly 24 nt sRNAs and mediating transcriptional silencing through epigenetic modifications, while other AGOs bind 21 nt sRNAs and mediate post-transcriptional gene silencing (Wang et al., 2011; Zhang et al., 2016). *Ph. infestans* has two AGOs belonging to clade 1 (*Ago1*/*2*) and three belonging to clade 2 (*Ago3-5*) (Vetukuri et al., 2011), while the *Phytophthora* spp. analyzed in this study have only one clade 1 and three to five clade 2 AGOs. The relatedness of the *Phytophthora* AGOs, and the distance to the lower number of more diverse AGOs in *Pythium* and *L. giganteum*, suggests that the *Phytophthora* sequences have undergone diversification since the split from other *Pythiaceae*. It remains to be determined how this relates to functioning of RNA silencing in *Pythium* and *L. giganteum*, but may explain the different abundances of different sRNAs sizes, especially those of 25/26 nt.

It was expected that the *Phytophthora* spp. would have major groupings of sRNAs at 21 nt and 25/26 nt. While a third group of sRNAs of 32 nt in length was observed in the work of Vetukuri et al., (2012) with *Ph. infestans*, we could not observe a peak at exactly this length for any *Phytophthora* spp. investigated in this study. However, there was a clear peak at 31 nt in each of the *Phytophthora* spp., and elevated numbers of 32 nt sRNAs were present in both *Py. oligandrum* and *Py. periplocum.* These longer sRNAs have been shown to be genuine in *Ph. infestans* and could be detected by Northern blot hybridisation analysis (Vetukuri et al., 2012).

*L. giganteum*, surprisingly, showed both a higher peak of 25–26 nt sRNAs and a more highly pronounced preference for 5′ uracil than the three *Phytophthora* spp., despite having an equal number of AGOs of clade 1 and 2. This could be due to the action of five RDRPs, a class of enzyme with only one or two members in all the other oomycetes analysed. It is possible that the combination of enzymes in *L. giganteum* preferentially produce sRNAs of 25–26 nt in length. The *L. giganteum* RDRPs are also peculiar in the fact that they are more similar to the ascomycetous *C. rosea* proteins than to the RDRPs of the other oomycetes. However, using BLAST we were able to identify similar RDRPs in other oomycete genera like *Aphanomyces*, *Achlya* and *Saprolegnia* (data not presented).

In accordance with previous studies, the sRNAs mapping to transposons and repeat-rich regions belonged mostly to the 25/26 nt size class, reinforcing the hypothesis that oomycetes use this class of RNAs to control transposons in their genomes (Åsman et al., 2016; Jia et al., 2017). However, in all species except *Ph. cactorum* and *L. giganteum* a second peak is present at 21 nt. Simple repeats are often the origin of 21 bp sRNAs in *Ph. infestans* (Fahlgren et al., 2013), and it is possible that they are the origin of the peak at this length for the species investigated here. Moreover, almost 50% of the repeat/transposon mapping sRNAs were of 31 nt in *Ph. cactorum*, a characteristic not shared by other *Phytophthora* species. Interestingly, this peak was unique to *Ph. cactorum* despite its similarity in repeat/transposon length and composition to *Ph. colocasiae*, that instead had a length distribution of sRNAs mapping to repeat/transposon closer to *Ph. plurivora*. This latter *Phytophthora* sp. had proportionally less Gypsy and other LTRs, more simple repeats and a total amount of repeats/transposons closer to *Pythium* species. This suggests that *Ph. cactorum* has a unique sRNA-dependent regulation mechanism based on sRNAs of 31 bp, which has not yet been explored. *Pythium* spp., on the other hand, had around six times less sRNAs mapping to repeat/transposon, in respect to *Phytophthora* spp., suggesting a functional difference in the role of RNA silencing among oomycetes.

*Clonostachys rosea*, the only non-oomycete included, had a very low amount of sRNA reads mapping to transposons despite having a similar quantity of them in its genome, compared with the analyzed oomycetes. Furthermore, most of the sRNAs mapping to *C. rosea* transposons were 18, 19, 20 or 23 nt in length, suggesting that fungi control transposons and repeat-rich genomic regions in different ways to oomycetes.

Oomycete genomes are characterized by having islands of densely packed genes, separated by long tracts of repeat-rich and gene sparse sequence. Oomycete gene promoters are very compact, and promoter regions frequently overlap on opposite DNA strands (Roy et al., 2013). Similarly, 3′ ends of genes can also be very close, and possibly overlap. The same study, which focused on *Ph. infestans*, also revealed that neighbouring genes were typically not activated at the same lifecycle stage, including those that were transcribed in antisense from small intergenic promotors. This may be a strategy that has evolved in oomycetes to regulate the transcriptional activity of densely packed gene space, while avoiding heterochromatin formation from RNA silencing mechanisms. Antisense overlapping regions were also a source of 25/26 nt sRNAs, even if a second peak was observable at 21 nt for all the oomycete species tested. The origin of some 21 nt sRNAs from the overlapping region of two adjacent genes has already been observed by Jia et al., (2017) in *Ph. parasitica*. In the current study, the species with the greatest extent of antisense overlapping regions were those with more sRNAs mapping to them, even after normalizing according to region length. The only exception was *Ph. cactorum* which, although it had only 23 kb per Mb of genome constituted by antisense overlapping regions, against the 105 kb/Mb of *Py. oligandrum*, was the species with the highest proportional number of sRNAs mapping to them. It should be noted that, while sRNAs are generated from these genomic antisense regions, the number of sRNAs from these regions is dramatically lower than that found for transposons.

CRNs and RxLRs are two large classes of effectors associated with pathogenicity of *Phytophthora* spp. (Anderson et al., 2015; Amaro et al., 2017; Liu et al., 2019). It has been observed in *Ph. infestans* that CRN genes are the source of mainly 21 nt sRNAs, while RxLRs are associated with the mapping of 25–26 nt sRNAs (Vetukuri et al., 2012; Fahlgren et al., 2013). Among the identified CRNs, less than 10% were predicted to be secreted *via* a conventional signal peptide, endoplasmic reticulum-Golgi route. If the CRNs without a predicted signal peptide are indeed extracellular, it would suggest that this class of effectors are secreted *via* non-conventional secretion mechanisms. Considering all the predicted CRNs, we found that 21 nt sRNAs are the most likely to map to CRNs in *Ph. colocasiae*, as was observed in *Ph. infestans* (Vetukuri et al., 2012; Fahlgren et al., 2013), while 25 nt and 26 nt were the most likely sRNA classes in *Ph. plurivora* and 21 nt, 25 nt, 26 nt and 32 nt had around the same frequency in *Ph. cactorum*. This was after normalizing each sRNA count according to the frequency of that sRNA size, meaning that 21 nt were not the dominant class of sRNAs mapping to CRNs in *Ph. cactorum* and *Ph. plurivora* even after considering that there were less 21 nt sRNAs than 25–26 nt. This suggests a high variability in sRNA mapping to this effector class in *Phytophthora* spp., which is also confirmed by the results of Jia et al. in *Ph. parasitica* (Jia et al., 2017) in which 53% of CRNs were associated with 25–26 nt sRNAs and not 21 nt. In comparison, both secreted and non-secreted RxLRs were more associated with 25-26 nt sRNAs in all *Phytophthora* spp., confirming the results of previous studies (Vetukuri et al., 2012; Fahlgren et al., 2013).

All other protein classes normally considered to contain effectors (secreted CAZymes, secreted proteases and other secreted proteins) had on average more sRNAs mapping to them in *Pythium* spp. than in other oomycetes. Both *Pythium* spp. had more sRNAs mapping to putative effectors compared to typical genes, while in both *Phytophthora* spp. and *L. giganteum* this number was lower for putative effector coding genes than for other genes. This suggests that effector production is generally not regulated by RNA silencing in these species, outside of specific effector classes like CRNs. Furthermore, it was interesting to observe that *L. giganteum* had more than double the number of secreted proteases (386) than *Py. oligandrum* (168), which was the oomycete species with the second highest amount. This may reflect the fact that a dedicated array of proteases is necessary to efficiently penetrate the cuticle of the insect hosts infected by *L. giganteum*.

Many families of CAZymes in oomycetes are composed of multiple members that have arisen by gene duplication (Ospina-Giraldo et al., 2010; Horowitz and Ospina-Giraldo, 2015; Liang et al., 2020; Sabbadin et al., 2021). In general, glycoside hydrolase family 17 (GH17) β-1,3-glucosidases are involved in cell wall biogenesis and modification, which is a prerequisite for cell growth and development (Beauvais and Latgé, 2018). Moreover, CAZymes of this class have been observed to be highly expressed during the early, mid and late stages of lupin infection by *Ph. parasitica*, and in all life stages in *Ph. kernoviae* (Blackman et al., 2015; Wang et al., 2021). Their involvement in both infection and regular growth can be explained by the fact that oomycete cell walls contain a high proportion of β-1,3-glucans, and plant cell walls can contain β-1,3-glucans as callose in response to pathogen infection. The high proportion of sRNAs mapping to genes coding for this class of CAZymes suggests that the careful regulation of these genes is crucial for normal growth and development in oomycetes. The significant differences in proportion of sRNAs mapping to GH17 coding genes between *Phytophthora* spp. and *Pythium* spp. is probably due to the evolutionary history of this GH family in oomycetes. Gene expression regulation of certain genes of this class is also reported in the fungal plant pathogen *Cladosporium fulvum* during late-stage infection of its host plant (Ökmen et al., 2019). Ökmen et al. found that a GH17 enzyme was expressed only during the late-stage of infection. Its activity released a damage-associated molecular pattern (DAMP) which induced the hypersensitive response and consequent reduction of fungal growth if the gene was constitutively expressed. In this light, our results might suggest that RNA silencing contributes to the tight regulation of GH17 proteins in the analyzed oomycetes, allowing them to influence cell wall biosynthesis, degrade plant cell walls and affect the induction of defense in plant hosts, a phenomenon fundamental for the action of both pathogens and biocontrol organisms. In both *Py. periplocum* and *Py. oligandrum* GH17 CAZymes have also been observed to be differentially regulated during specific stages of the interaction with *Ph. infestans* (Liang et al., 2020), therefore these β-1,3-glucosidases likely have a role during host parasitism, further explaining the high amount of sRNAs mapping to them in the analyzed *Pythium* species.

Another class of CAZyme with a consistent number of sRNAs mapping to it was AA17, an oomycete-specific family which was consequently not identified in *C. rosea*. This class had the highest number of sRNAs mapping to it in *Phytophthora* spp. and *L. giganteum*, and it is formed of copper-dependent lytic polysaccharide monooxygenases which are the most-induced CAZymes during early stages of *Ph. infestans* infection of tomato (Sabbadin et al., 2021). In *Phytophthora* spp. these enzymes can facilitate host penetration by cleaving pectin and disrupting the plant cell wall network, while possibly interfering with host immunity (Sabbadin et al., 2021). It is possible that there are also other substrate specificities in this CAZyme family. In the *P. infestans* genome, AA17 proteins form clusters of closely related (likely duplicated) genes. The high amount of sRNA mapping to them in all the oomycetes analysed suggest that RNA silencing has a role in their regulation and importance, even in *Pythium* spp. and *L. giganteum*.

The remaining secreted CAZyme classes predicted to be most affected by RNA silencing were all involved in hemicellulose degradation, but they differed between individual species. GH30 (xylanases and glucosidases) and PL3 (pectate lyases) originated more sRNAs in *Ph. cactorum* and *Ph. colocasiae*, while GH3 (glucosidases, xylosidases and alpha-L-arabinofuranosidases) and CE8 (pectin methylesterase) were more represented in *Ph. plurivora*, suggesting different hemicellulose degradation capabilities in these species.

Novel milRNAs were predicted for all the species analysed, but only one was shared between different species (*Py. oligandrum* and *Py. periplocum*), suggesting that most milRNAs have a species-specific function in oomycetes. This would also explain why known *Ph. infestans* milRNA miR8788 (Hu et al., 2022) was not detected in our dataset. MilRNA pca_mir_1 of *Ph. cactorum* was predicted to target the transcript Pcac1_g11221.t1, coding for the RxLR effector KAG2778520.1. The homolog of this transcript in *Ph. cactorum* strain 10,300, called U17742, was observed to be upregulated in germinating cysts with germ tubes, compared to mycelium and zoospores, and it is therefore possible that milRNA pca_mir_1 could be involved in the fine-tuning of the infection process (Chen et al., 2018). During the prediction of cross-species targets, *Py. periplocum* milRNA Ppe_mir_2 was predicted to target *Ph. infestans* gene PITG_13437, coding for a metalloprotease of family M16C. Several metalloproteases have been characterized as virulence factors for *Ph. infestans* (Schoina et al., 2021), and the potential for targeting enzymes of this class by *Py. periplocum* milRNAs suggests the possibility for this oomycete to use cross-kingdom RNA silencing as a component of its biocontrol action. The same milRNA was also predicted to target four *B. cinerea* transcripts with no predicted role in disease induction, and therefore it could have a function in transboundary RNA silencing against this fungal plant pathogen.

The prediction of putative cross-species targets was also performed for *L. giganteum* and its mosquito host *A. aegypti,* but animal-based target prediction software programs often search only for a 2–9 nt complementarity region and they produce a high number of false positives (Peterson et al., 2014; Riffo-Campos et al., 2016). In this case we predicted 5,227 putative *A. aegypti* targets for the milRNAs of *L. giganteum*, and expression, localization and binding analyses are necessary to reduce this number.

## Conclusion

Oomycete species cause disease on a wide variety of host organisms. Their pathogenicity arsenal includes many protein effectors and can also include trans-boundary RNA silencing. To date, the proteins that generate sRNAs, and sRNA populations, have only been explored in a few species of *Phytophthora*. Here we extended the survey of RNA silencing proteins and sRNAs in three more species of *Phytophthora*, two *Pythium* spp., and *L. giganteum*. Our results show that, while all the species surveyed contain a single DCL protein, there was greater diversity in AGO and RDRP proteins that may be reflected in the sRNA populations. The major role for RNA silencing in the oomycetes appears to be for controlling the expansion of mobile elements in their genomes, but further roles may also be to limit gene expression from duplicated copies of genes, and regulation of effector gene expression, a process which seems more frequent in *Pythium* spp. than other oomycetes. Moreover, specific gene families seem to be targeted by precise sRNA sizes, such as effectors of the RxLR class which are predominantly targeted by 25/26 nt sRNAs in *Phytophthora* spp. The discovery of novel milRNAs from the biocontrol species *Py. periplocum* and *L. giganteum* with putative gene targets in hosts reveals the potential role for trans-boundary sRNA transport during biocontrol interactions.

## Data availability statement

The datasets presented in this study can be found in online repositories. The names of the repository/repositories and accession number(s) can be found in the article/Supplementary material.

## Author contributions

RV, MD, and SW planned the study. RV prepared samples for sRNA sequencing. EP performed sRNA data analysis. EP, BK, and PS performed the phylogenetic analyses. EP wrote the first draft of the manuscript. RV and MD secured the funding. All authors contributed to the article and approved the submitted version.

## Funding

This work was financially supported by the Swedish Research Council for Environment, Agricultural Sciences and Spatial Planning (FORMAS; grant numbers 2018–01420 and 2019–01316), Novo Nordisk Fonden (0074727), the Swedish Research Council (2019–04270), and Carl Tryggers Stiftelse för Vetenskaplig Forskning (CTS 19: 82, CTS 20: 464).

## Conflict of interest

The authors declare that the research was conducted in the absence of any commercial or financial relationships that could be construed as a potential conflict of interest.

## Publisher’s note

All claims expressed in this article are solely those of the authors and do not necessarily represent those of their affiliated organizations, or those of the publisher, the editors and the reviewers. Any product that may be evaluated in this article, or claim that may be made by its manufacturer, is not guaranteed or endorsed by the publisher.

## Footnotes

^1^ https://github.com/EdoardoPiombo/promoter_extractor

^2^ https://github.com/dpryan79/Answers/tree/master/bioinfoSE_3181
