# Supplementary figures and images for: RNA silencing proteins and small RNAs in oomycete plant pathogens and biocontrol agents
Source: Front Microbiol. 2023 Mar 24;14:1076522. doi: 10.3389/fmicb.2023.1076522 (PMC10080066; doi:10.3389/fmicb.2023.1076522)

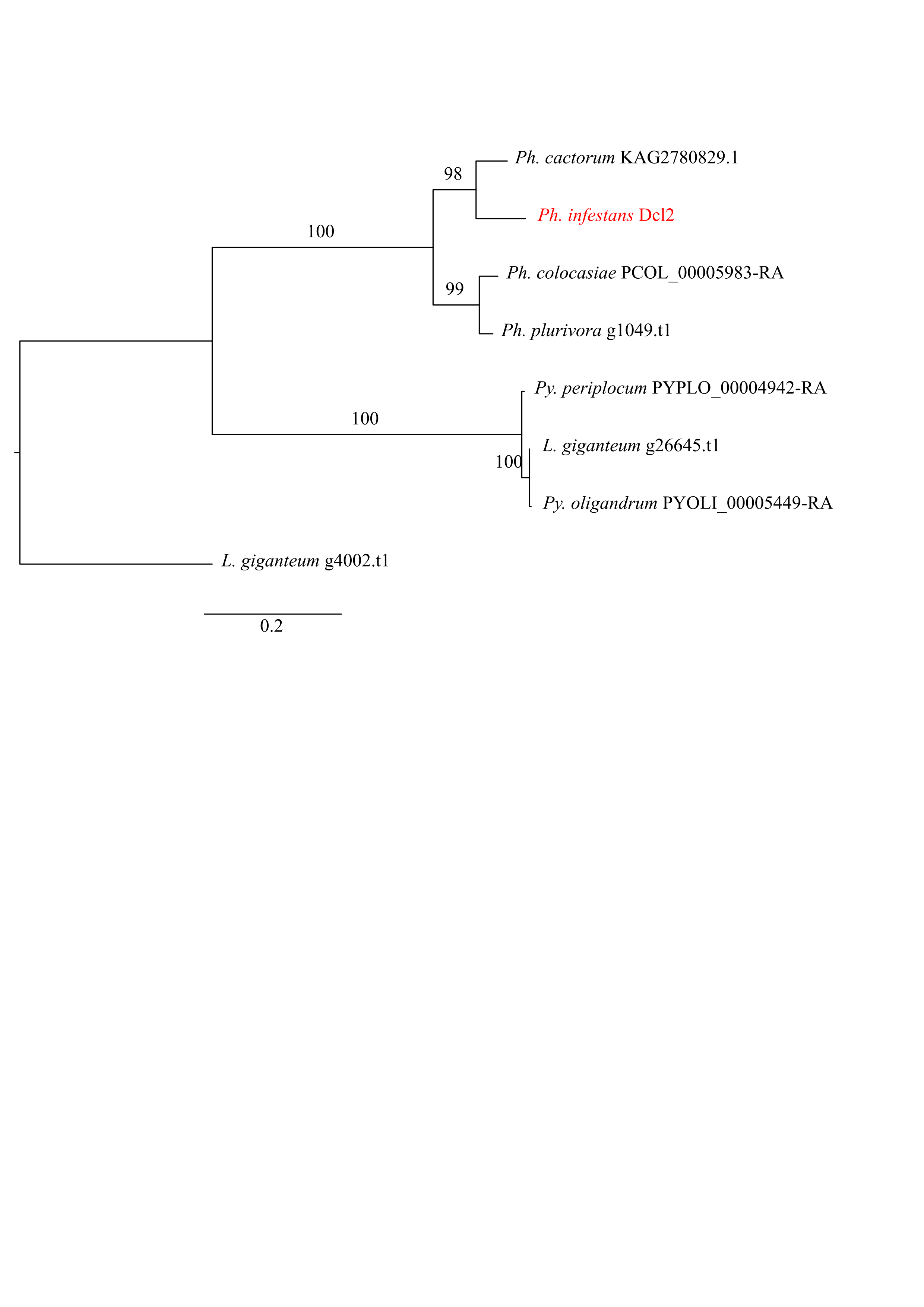

Supplement: Supplementary file 3 [file Image_1.TIFF]
